# Supplementary material for: Adipocytes Promote Breast Cancer Cell Survival and Migration through Autophagy Activation
Source: Cancers (Basel). 2021 Aug 3;13(15):3917. doi: 10.3390/cancers13153917 (PMC8345416; doi:10.3390/cancers13153917)
Supplement: Supplementary file 1 [file cancers-13-03917-s001.zip › cancers-1273104-supplementary.pdf]

Article

Adipocytes Promote Breast Cancer Cell Survival and Migration through Autophagy Activation

Dorine Bellanger, Cléa Dziagwa, Cyrille Guimaraes, Michelle Pinault, Jean-François Dumas and Lucie Brisson *

Table S1. Primers used during qPCR assay.

Primers	Forward Primer	Reverse Primer
muPerilipin1	CTCTGGGAAGCATCGAGAAG	GCATGGTGTGTCGAGAAAGA
muFABP4	CCACCATAAAGAGAAAACGAGAG	GTGGAAGTGACGCCTTTCAT
muIRF1	CAGACGAGGATGAGGAAGGG	GAGGGCTGTCAATCTCTGGT
muFRK	GGTTGGATGAAGGTGGCTTC	CGGTCTATCTCCCACTGGTC
muGSTM5	GCACAACATGTGTGGTGACA	CTGTGCAGGTAGCTGTTCCA
muBcL2L11	CAACACAAACCCCAAGTCCT	CATTTGCAAACACCCTCCTT
muRAD52	AGTTTTGGGAATGCACTTGG	TCGGCAGCTGTTGTATCTTG
muHPRT	TGGGCTTACCTCACTGCTTT	CTAATCACGACGCTGGGACT
muActB	TTGGGTATGGAATCCTGTGG	GCACTGTGTTGGCATAGAGG

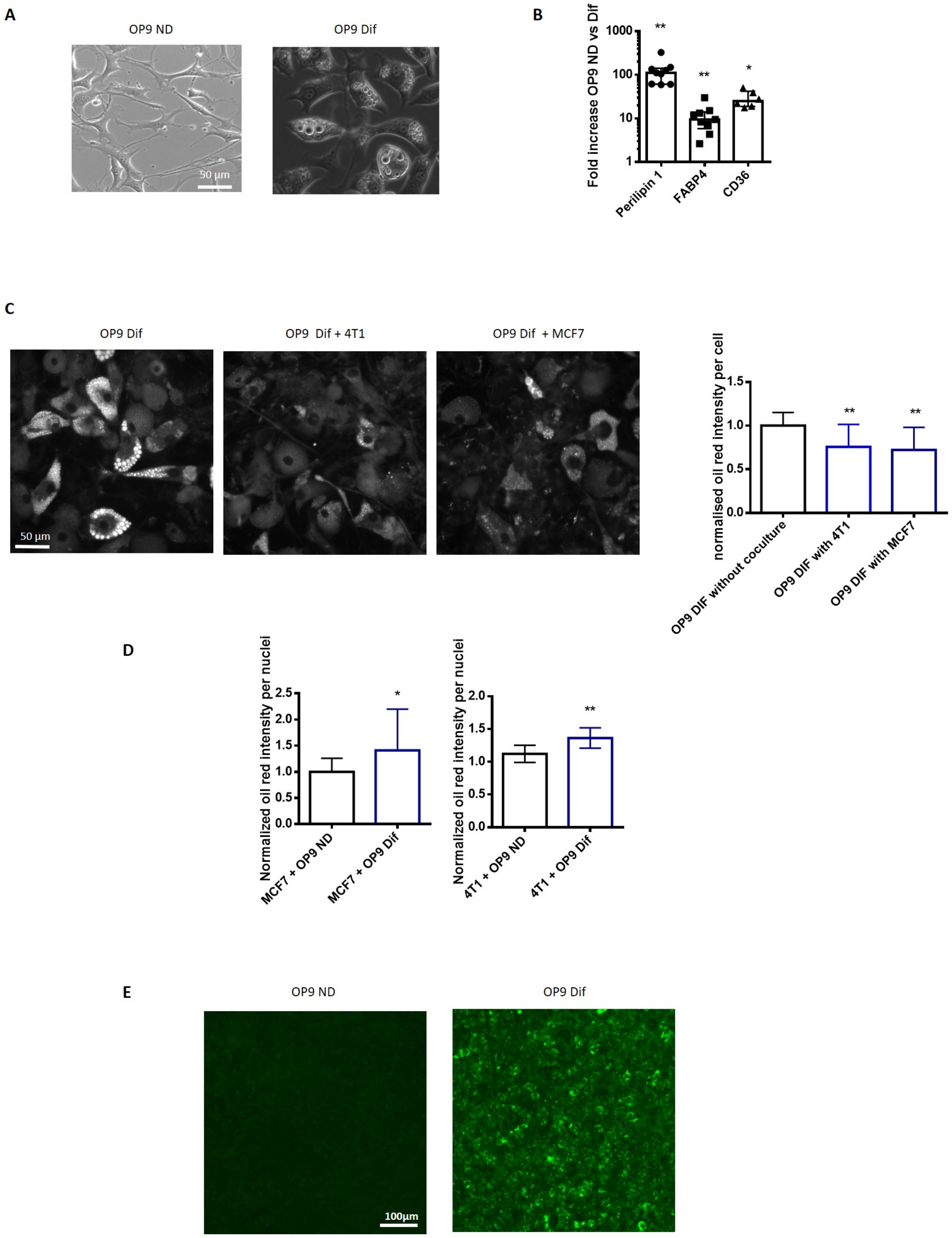

Figure S1. Differentiation of OP9 murine stem cells. (A) Representative images of OP9 murine stem cells ND and Dif. (B) mRNA quantification of adipocyte markers (Perilipin 1, FABP4, CD36) in OP9 Dif vs. OP9 ND. HPRT and β-actin were used as reference genes (Median ± interquartile range, Wilcoxon Signed Rank Test ns, n = 6–9). (C) Lipid droplet staining using Oil red O in Dif OP9 in co-culture with 4T1 and MCF7 cells (Median ± interquartile range, Kruskal–Wallis followed by Dunn's Multiple Comparison Test, n = 6). (D) Quantification of Figure 1A,B (Median ± interquartile range, Wilcoxon Signed Rank Test, n = 6). (E) OP9 ND and OP9 Dif were incubated with BODIPY FL C_{16} fluorescent fatty acids for 5 h (n = 5).

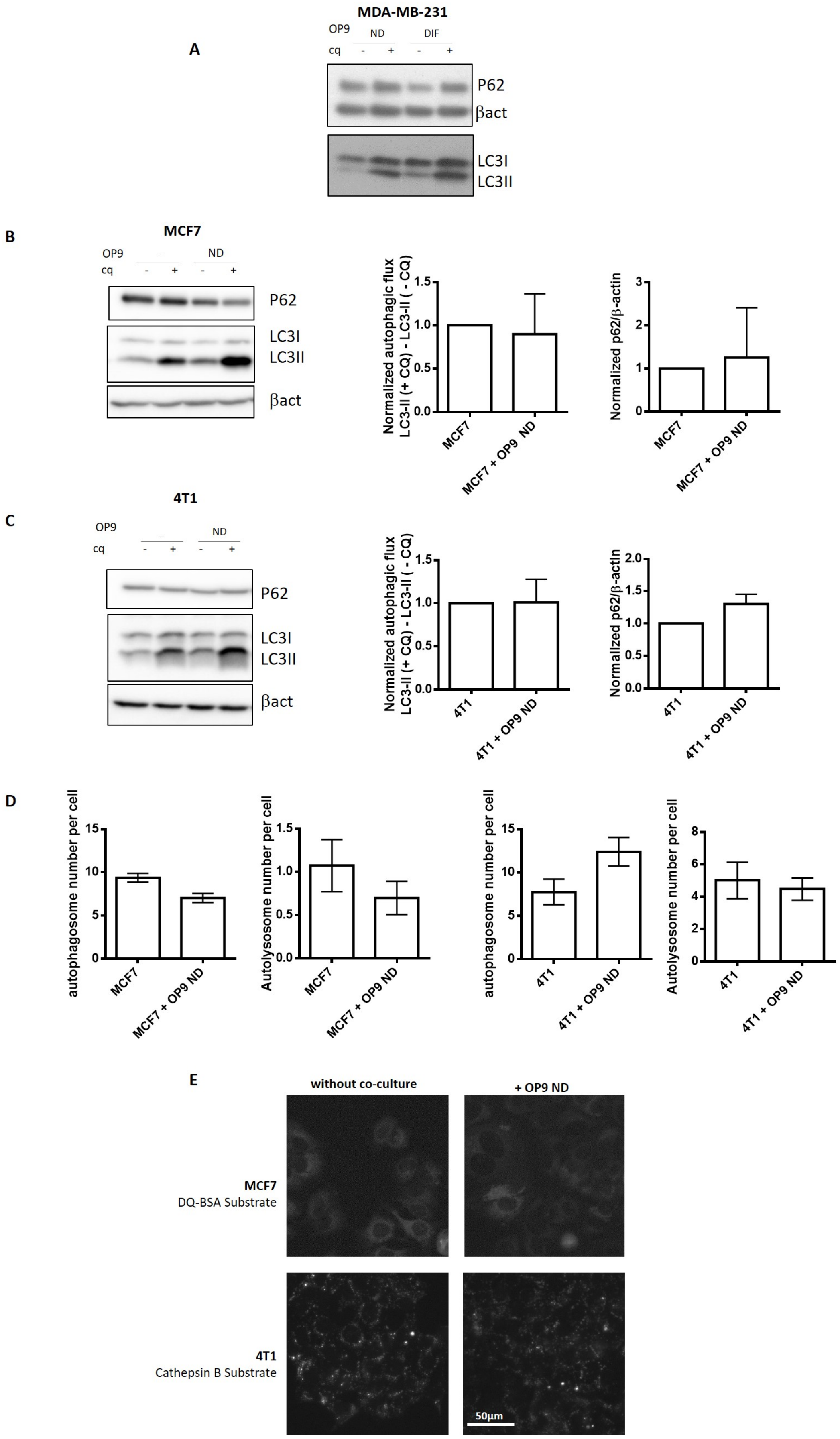

Figure S2. Co-culture with OP9 ND and OP9 Dif in breast cancer cells. (A) Representative western blots of LC3 and p62 protein expression in MDA-MB-231 cells in co-culture with OP9 ND and Dif for 48 h with 20 μM chloroquine (CQ) for 18 h. (B,C) Representative western blots and quantification of

normalised autophagic flux and p62 protein expression in MCF7 (B) and 4T1 (C) cells in co-culture with OP9 ND and without co-culture for 48 h. To calculate the autophagic flux, 20 μM chloroquine (CQ) was used for 18 h (Median ± interquartile range, Wilcoxon Signed Rank Test, n=5-6, ns). (D) Autophagosome and autolysosome number was measured using the LC3-GFP-mRFP plasmid in MCF7 and 4T1 cells in co-culture with OP9 ND and without co-culture for 48 h. DAPI was used to normalise by cell number (Median ± interquartile range, Kruskal–Wallis test followed by Dunn's Multiple Comparison Test, *n* = 3, ns). (E) Intracellular degradation, measured in MCF7 cells using DQ-BSA substrate, and in 4T1 cells using Cathepsin B substrate, in cells in proximity to OP9 ND compared to without co-culture (*n* = 3).

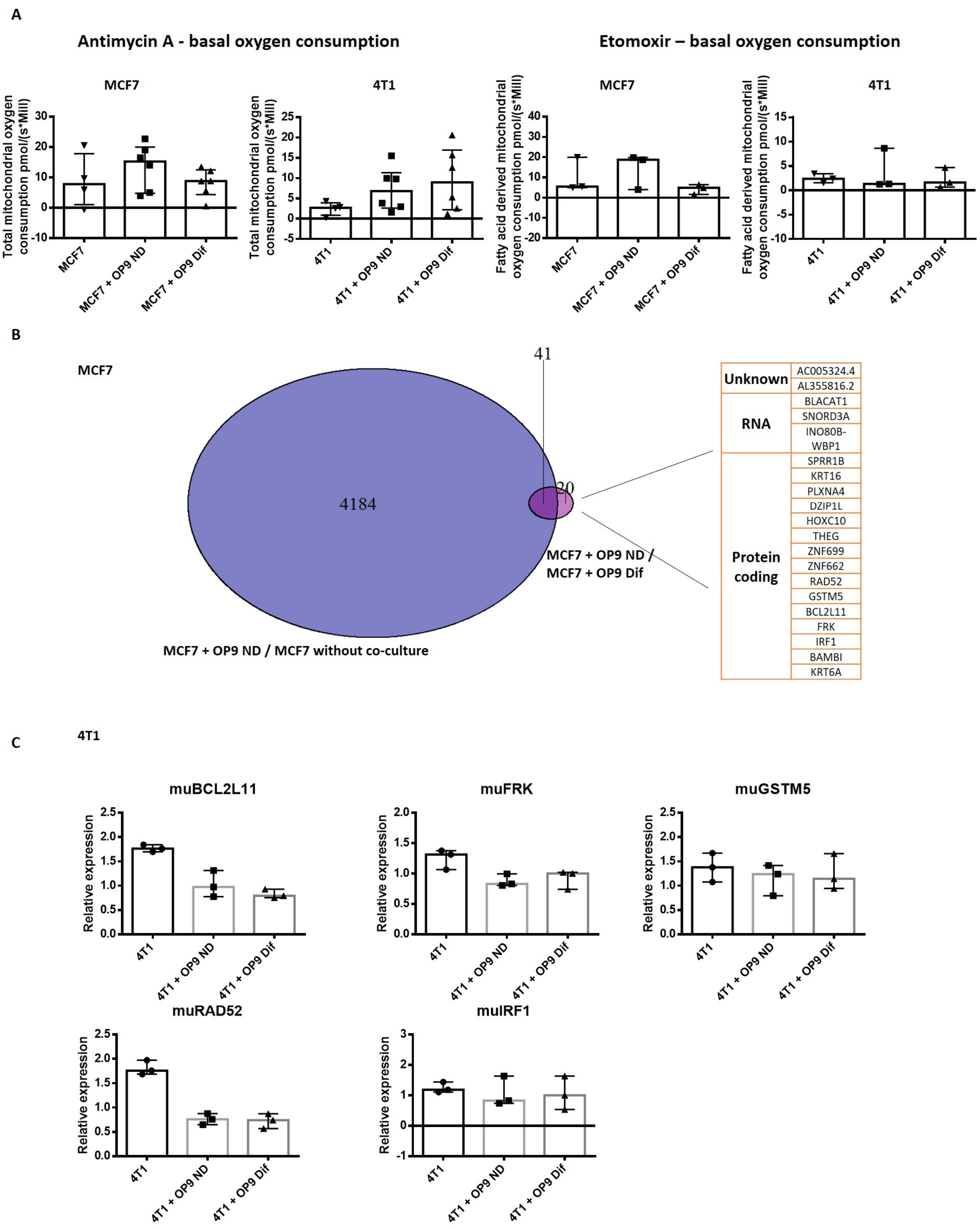

Figure S3: Adipocytes do not modify mitochondrial oxidation and transcriptional regulation. (A) Total mitochondrial oxygen consumption (left) and fatty acid-derived mitochondrial oxygen

consumption (right) in MCF7 and 4T1 cells, without co-culture and in co-culture with OP9 ND and OP9 Dif (Median ± interquartile range, Mann–Whitney test OP9 ND vs. OP9 dif ns, n = 3–6). (B) Venn diagram of RNA sequencing performed in MCF7 cells without co-culture and in co-culture with OP9 ND and OP9 Dif. Genes specifically regulated in conditions of co-culture with OP9 Dif vs. OP9 ND are presented on the right (n = 3). (C) mRNA expression of BCL2L11, FRK, GSTM5, RAD52 and IRF1 in 4T1 cells without co-culture and in co-culture with OP9 ND and OP9 Dif. HPRT and β-actin were used as reference genes (Median ± interquartile range, Mann–Whitney test OP9 ND vs. OP9 dif ns, n = 3.

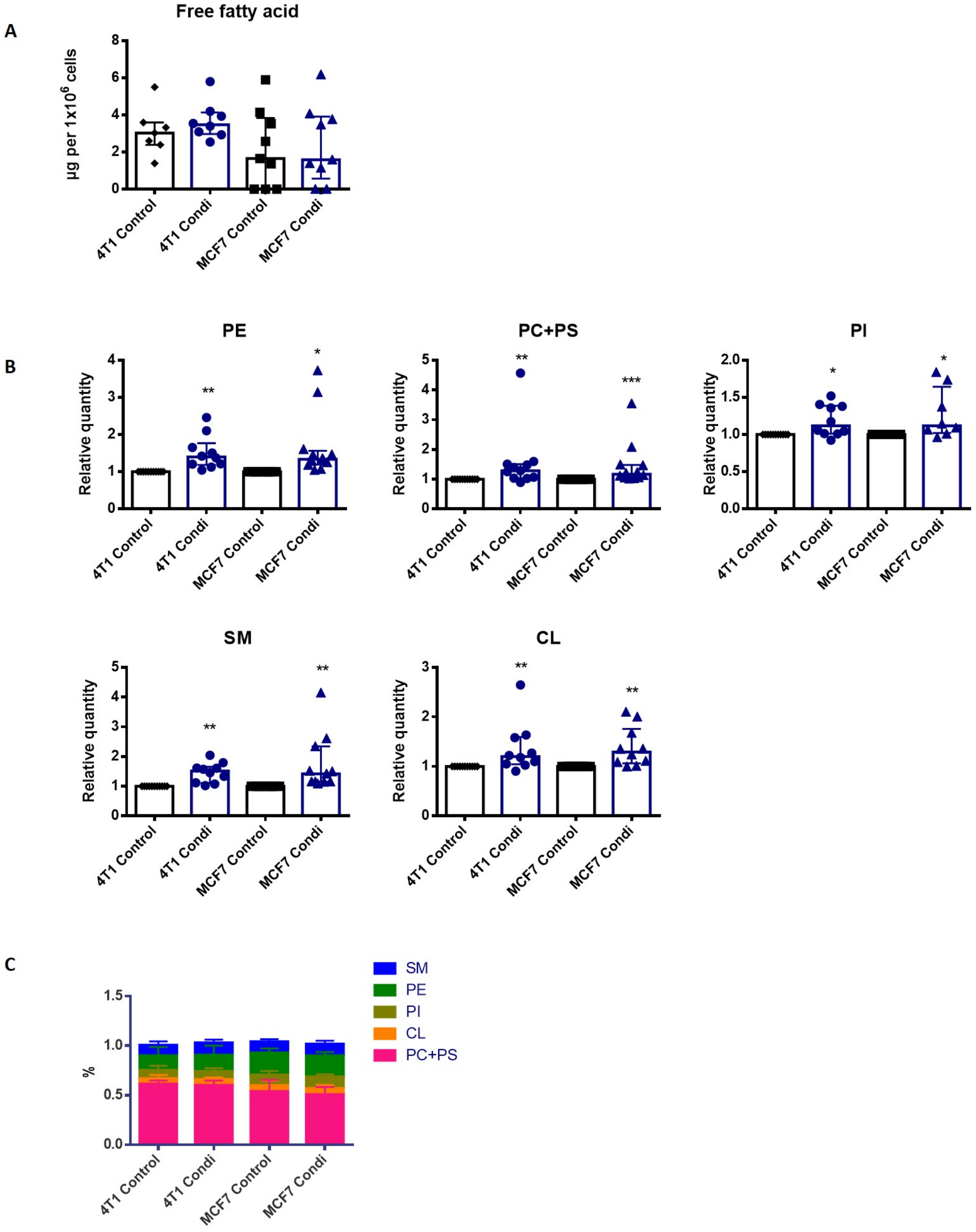

Figure S4. Lipid species in breast cancer cells treated with adipocyte conditioned media. (A) Free fatty acids measured by HPTLC in MCF7 and 4T1 cells treated with control and conditioned media from OP9 Dif for 24 h (Median ± interquartile range, Mann–Whitney test ns, n = 8). (B) Phospholipid classes: sphingomyelin SM, phosphatidylcholine PC, phosphatidylserine PS, phosphatidylinositol PI,

phosphatidylethanolamine PE, cardiolipin CL, measured by HPTLC in MCF7 and 4T1 cells treated with control and conditioned media from OP9 Dif for 24 h (Median ± interquartile range, Wilcoxon Signed Rank Test, n = 10–12). (C) Representation in percentage of data in B (two-way ANOVA followed by Bonferroni's multiple comparisons test.

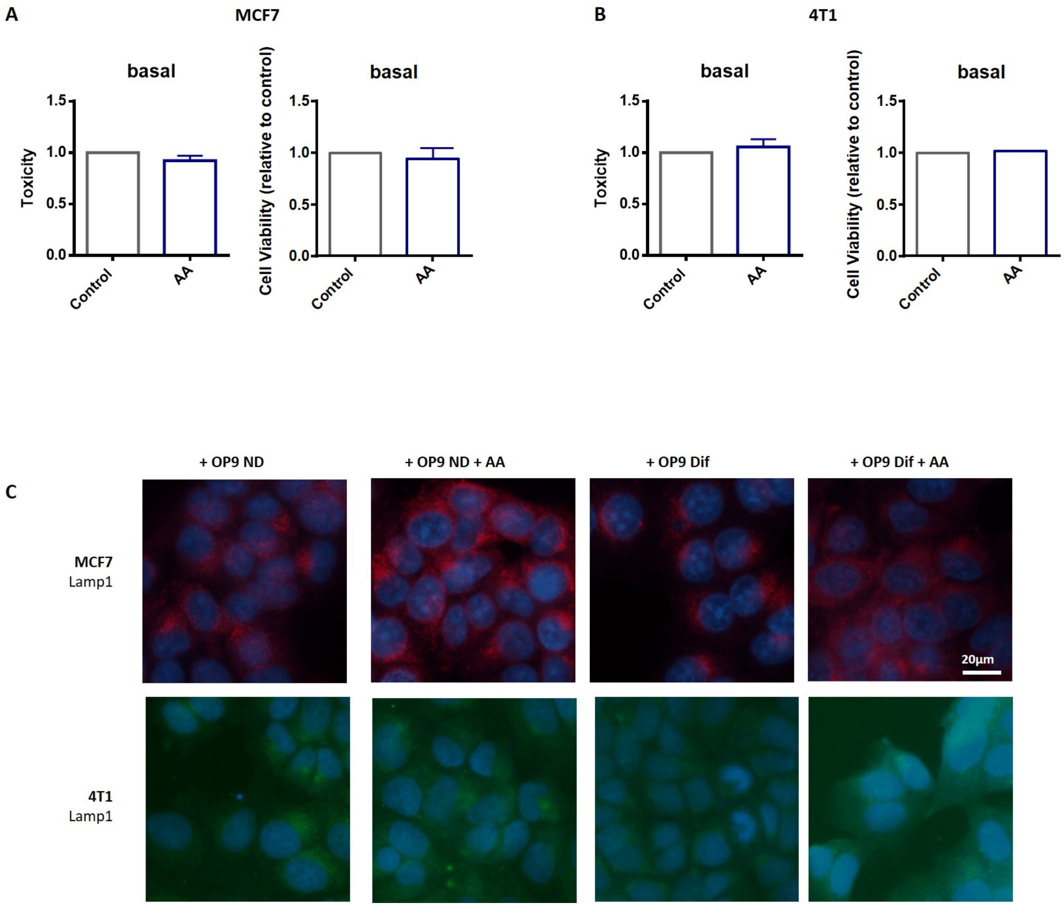

Figure S5: Arachidonic acid supplementation in breast cancer cells. (A,B) Toxicity and cell viability assays in MCF7 (A) and 4T1 (B) cells treated with 20 µM of arachidonic acid (AA) for 24 or 48 h (Median ± interquartile range, Wilcoxon Signed Rank Test, n = 3). (C) Lysosomes were stained in MCF7 and 4T1 cells in co-culture with OP9 ND and OP9 Dif, and treated with and without

arachidonic acid (AA) 20 µM for 48 h

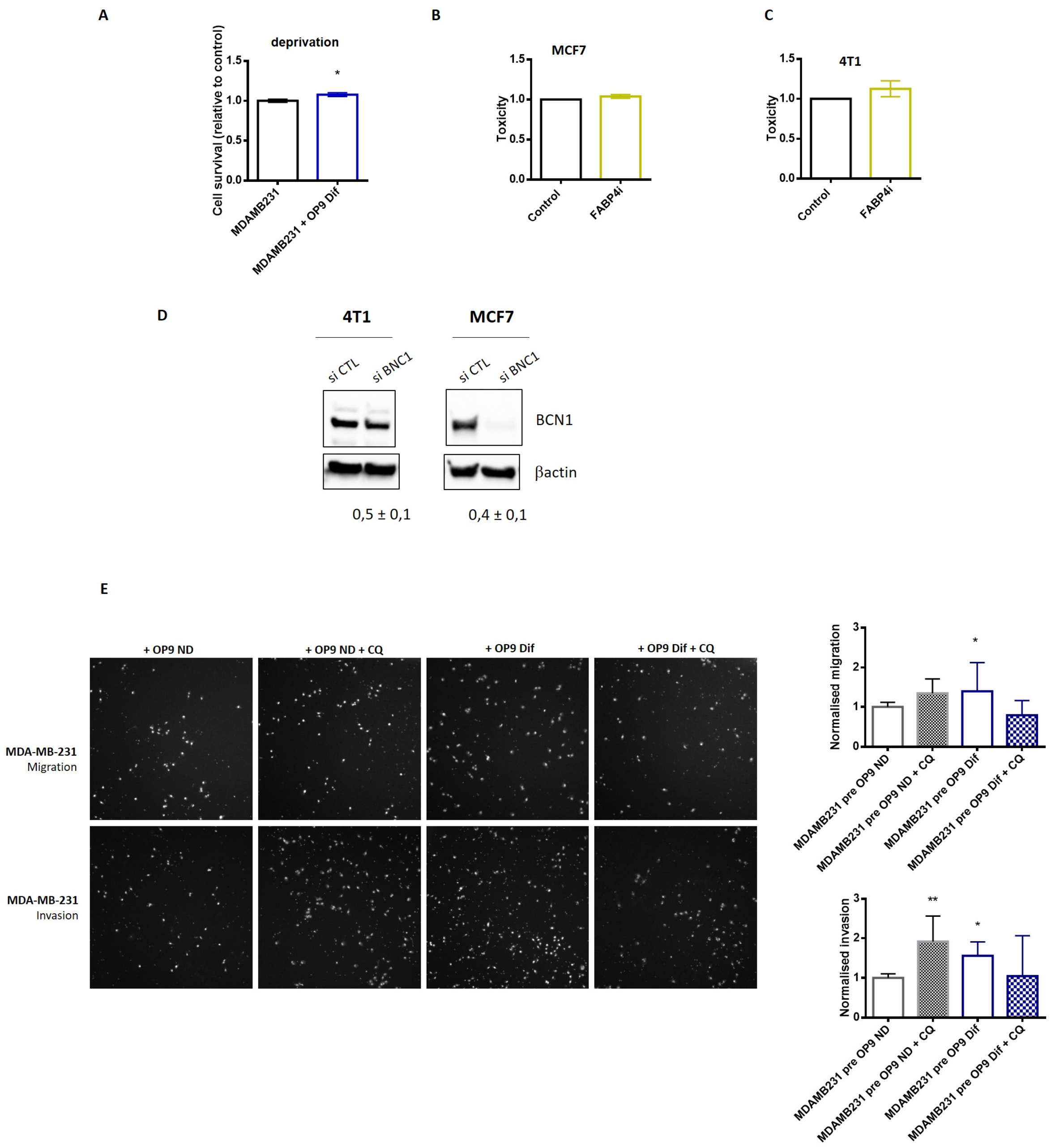

Figure S6: FABP4 inhibition, siBCN1and co-culture with OP9 Dif in breast cancer cells. (A) Cell survival assay in MDA-MB-231 cells with and without OP9 Dif in HBSS deprivation medium for 48 h (Mean ± SEM, One sample *t* test, *n* = 5). (B,C) Toxicity assay in MCF7 (B) and 4T1 (C) cells treated with 20 µM of FABP4 inhibitor for 24 h (Median ± interquartile range, *n* = 2). (D) Representative western blot for the efficiency of siRNA against Beclin1 (siBCN1) in 4T1 and MCF7 cells. Percentage of Beclin 1 expression in siBCN1 compared to siRNA control (siCTL) is indicated below (Mean ± SEM, *n* = 8). (E) MDA-MB-231 cells were co-cultured with OP9 ND and OP9 Dif for 48 h and then subjected to cell migration and invasion assays with and without chloroquine (CQ) 20 µM for 24 h (Median ± interquartile range, Wilcoxon Signed Rank Test, *n* = 12).
